# Tectal glioma: clinical, radiological, and pathological features, and the importance of molecular analysis

**DOI:** 10.1007/s10014-024-00494-9

**Published:** 2024-10-21

**Authors:** Ryoji Imoto, Yoshihiro Otani, Kentaro Fujii, Joji Ishida, Shuichiro Hirano, Naoya Kemmotsu, Yasuki Suruga, Ryo Mizuta, Yasuhito Kegoya, Yohei Inoue, Tsuyoshi Umeda, Madoka Hokama, Kana Washio, Hiroyuki Yanai, Shota Tanaka, Kaishi Satomi, Koichi Ichimura, Isao Date

**Affiliations:** 1 Department of Neurological Surgery, Okayama University Graduate School of Medicine, Dentistry and Pharmaceutical Sciences, 2-5-1 Shikata-cho, Kita-ku, Okayama, 700-8558 Japan; 2 Department of Brain Disease Translational Research, Juntendo University Graduate School of Medicine, 2-1-1 Hongou, Bunkyou-ku, Tokyo, 113-8421 Japan; 3 Department of Pediatrics, Okayama University Graduate School of Medicine, Dentistry and Pharmaceutical Sciences, 2-5-1 Shikata-cho, Kita-ku, Okayama, 700-8558 Japan; 4 Department of Pathology, Okayama University Hospital, 2-5-1 Shikata-cho, Kita-ku, Okayama, 700-8558 Japan; 5 Department of Pathology, Kyorin University Faculty of Medicine, 6-20-2 Shinkawa, Mitaka-shi, Tokyo, 181-8611 Japan

**Keywords:** Tectal glioma, Lower grade glioma, *KRAS*, H3 K27M, Molecular analysis

## Abstract

Tectal glioma (TG) is a rare lower grade glioma (LrGG) that occurs in the tectum, mainly affecting children. TG shares pathological similarities with pilocytic astrocytoma (PA), but recent genetic analyses have revealed distinct features, such as alterations in *KRAS* and *BRAF*. We conducted a retrospective review of cases clinically diagnosed as TG and treated at our institute between January 2005 and March 2023. Six cases were identified and the median age was 30.5 years. Four patients underwent biopsy and two patients underwent tumor resection. Histological diagnoses included three cases of PA, one case of astrocytoma, and two cases of high-grade glioma. The integrated diagnosis, according to the fifth edition of the World Health Organization Classification of Tumours of the central nervous system, included two cases of PA and one case each of diffuse high-grade glioma; diffuse midline glioma H3 K27-altered; glioblastoma; and circumscribed astrocytic glioma. Among the three patients who underwent molecular evaluation, two had *KRAS* mutation and one had *H3-3A* K27M mutation. Our results demonstrate the diverse histological and molecular characteristics of TG distinct from other LrGGs. Given the heterogeneous pathological background and the risk of pathological progression in TG, we emphasize the importance of comprehensive diagnosis, including molecular evaluation.

## Introduction

Tectal glioma (TG) is a rare glioma originating in the dorsal part of the midbrain, consisting of the superior and inferior colliculi, occurring predominantly in children [8, 12]. The clinical course is generally indolent and tends to present with neurological symptoms such as intracranial pressure increases due to hydrocephalus obliterans. The most common symptoms are headache, gait disturbance, and ataxia related to hydrocephalus obliterans, along with visual field deficits and cognitive dysfunction [8, 10, 12]. TG is typically classified as pilocytic astrocytoma (PA) or lower grade astrocytic glioma, and tends to have a good prognosis [12]. However, Mohme et al. [19] reported that subpopulations of TGs may undergo malignant transformation in children and adolescents. The pathogenesis of TG remains largely unknown. With the recent progress of molecular analysis, *KRAS* G12R, *BRAF* V600E mutation, and *KIAA1549::BRAF* fusion were identified in TG, and the DNA methylation profile of TG suggests its classification as a distinct entity from other lower grade glioma (LrGG)s [6, 12]. In addition, several cases of TG harboring the H3 K27M mutation have been reported, despite histologically resembling PA with pleomorphism [20, 25]. Thus, TG exhibits a unique clinical course, histology, and molecular profile, and its pathogenesis remains unknown. Therefore, we conducted a retrospective review of patients clinically diagnosed with TG at our institution, examining their clinical course, imaging findings, histological features, and molecular evaluations to gain insight into the pathological background of TG.

## Materials and methods

### Patient cohort

We retrospectively included patients with radiographically suspected TG and pathologically diagnosed cases between January 2005 and March 2023. All patients underwent surgery, and those with pineal lesions or tumors extending upward into the upper brainstem from the pons or downward from the thalamus were excluded from the study. Chart review was used to collect clinical characteristics, clinical course, imaging findings, and histological findings. For image analysis, we assessed tumor size on T2-weighted images and the ratio of cystic and enhancing tumor components on T1-gadolinium images using magnetic resonance imaging (MRI). Tumor progression was radiologically defined using Response Assessment in Neuro-Oncology (RANO) criteria. This retrospective study received approval from the Institutional Review Board of Okayama University Hospital, and written informed consent was provided from all patients (1911–023).

### Pathological analysis

The histopathology of all cases was reviewed by a board-certified pathologist, with pathological diagnoses made according to the fourth edition of the World Health Organization (WHO) Classification of Tumours of the central nervous system (CNS). Hematoxylin and eosin (H&E) and immunohistochemical staining were examined using an Olympus BX53 light microscope (Olympus, Tokyo, Japan). For immunohistochemical staining, the following antibodies were used: anti-H3 K27M antibody (EMD Millipore, ABE419, diluted 1:1000), anti-H3 K27me3 antibody (Cell Signaling Technology, #9733, diluted 1:400), and anti-Ki67 antibody (Agilent Dako, 41664650, diluted 1:50). Antigen retrieval was performed by autoclaving for 15 min at 121 °C in citrate buffer.

### Molecular analysis

Regarding genome-wide DNA methylation, genomic DNA was extracted from frozen tissue from two TG samples and analyzed using the German Cancer Research Center (DKFZ) methylation profiling classifier (DKFZ classifier), as previously described [4, 26].

FoundationOne^®^ CDx (F1CDx) is another comprehensive genomic profiling tool that analyzes the exons of 324 genes and introns of 36 genes, which are frequently altered in various solid tumors [16, 18, 29]. F1CDx was performed on two samples of DNA extracted from formalin-fixed paraffin-embedded tissue.

## Results

### Clinical features

Six cases were identified; five were female and one was male. Patient characteristics are summarized in Table 1. The median age at diagnosis was 30.5 years (range: 6–45 years), and two of the six patients were pediatric cases. Clinical symptoms due to intracranial hypertension were observed in most patients, including headache in four patients, and gait disturbance and oculomotor nerve palsy in three patients each. Diplopia was observed in two patients, and vomiting, ataxia, and cognitive decline in one patient each. The mean tumor diameter was 22.8 mm (range: 14.7–33.2 mm). All six patients had enlarged ventricles, with contrast enhancement of the tumor in four cases and cyst formation in two cases. The median duration from discovery to treatment was 17.5 days (range: 6–66 days). Four patients underwent biopsy or biopsy with third ventriculostomy, and two patients underwent tumor resection. Three patients received radiotherapy and two received chemotherapy as adjuvant therapy after primary surgery.

**Table 1:** Clinical characteristics of six tectal gliomas

Case	Age (yr)	Sex	Surgical procedure	Histological diagnosis^a^	Integrated diagnosis^b^	Molecular analysis^c^	DKFZ classifier v11b4^c^ (calibrated score)	Radiographical findings (MRI)				Adjuvant therapy		PFS (yr)	OS (yr)	Outcome
								Size^d^ (mm)	Contrast enhancement	Ventricular enlargement	Cystic formation	Radio-therapy	Chemo-therapy			
1	6	F	Biopsy + ETV → Cyst resection + reservoir placement	Pilocytic astrocytoma	Pilocytic astrocytoma	N/A	N/A	19.5	–	+	–	+	–	3.8	6.7^e^	A
2	14	M	Biopsy + ETV → Resection	Glioblastoma, IDH-wild type	Diffuse high-grade glioma, NEC	*KRAS* G12A *KRAS* G13R	Pilocytic astrocytoma (0.71)	22.7	+	+	–	+	+	1.4	1.4	D
3	40	F	Biopsy + ETV	Pilocytic astrocytoma	DMG H3 K27-altered	H3 K27M	DMG H3 K27M mutation (0.93008)	16.4	–	+	–	–	–	4.5	7.2^e^	A
4	45	F	Biopsy	Pilocytic astrocytoma	Pilocytic astrocytoma	N/A	N/A	14.7	+	+	–	–	–	7.3^e^	7.3^e^	A
5	36	F	Biopsy → Resection	Anaplastic astrocytoma	Glioblastoma, NOS	N/A	N/A	33.2	+	+	+	+	+	2.5	5.6	D
6	25	F	Biopsy → ETV + reservoir placement	Astrocytoma grade II	Circumscribed astrocytic glioma, NOS	*KRAS* Q61R	N/A	30.2	+	+	+	–	–	2.9^e^	2.9^e^	A

*A* alive, *D* dead, *DMG* diffuse midline glioma, *ETV* endoscopic third ventriculostomy, *F* female, *M* male, *NEC* not elsewhere classified, *N/A* not available, *NOS* not otherwise specified, *OS* overall survival, *PFS* progression-free survival *yr*: year

^a^The diagnosis according to the fourth edition of the WHO Classification of Tumours of the CNS

^b^The diagnosis according to the fifth edition of the WHO Classification of Tumours of the CNS

^c^Genome-wide DNA methylation profiling was performed in Case 2 and 3. FoundationOne CDx was performed in Case 2 and 6

^d^The maximum diameter of the tumor

^e^Censored for survival

**Fig. 1:**
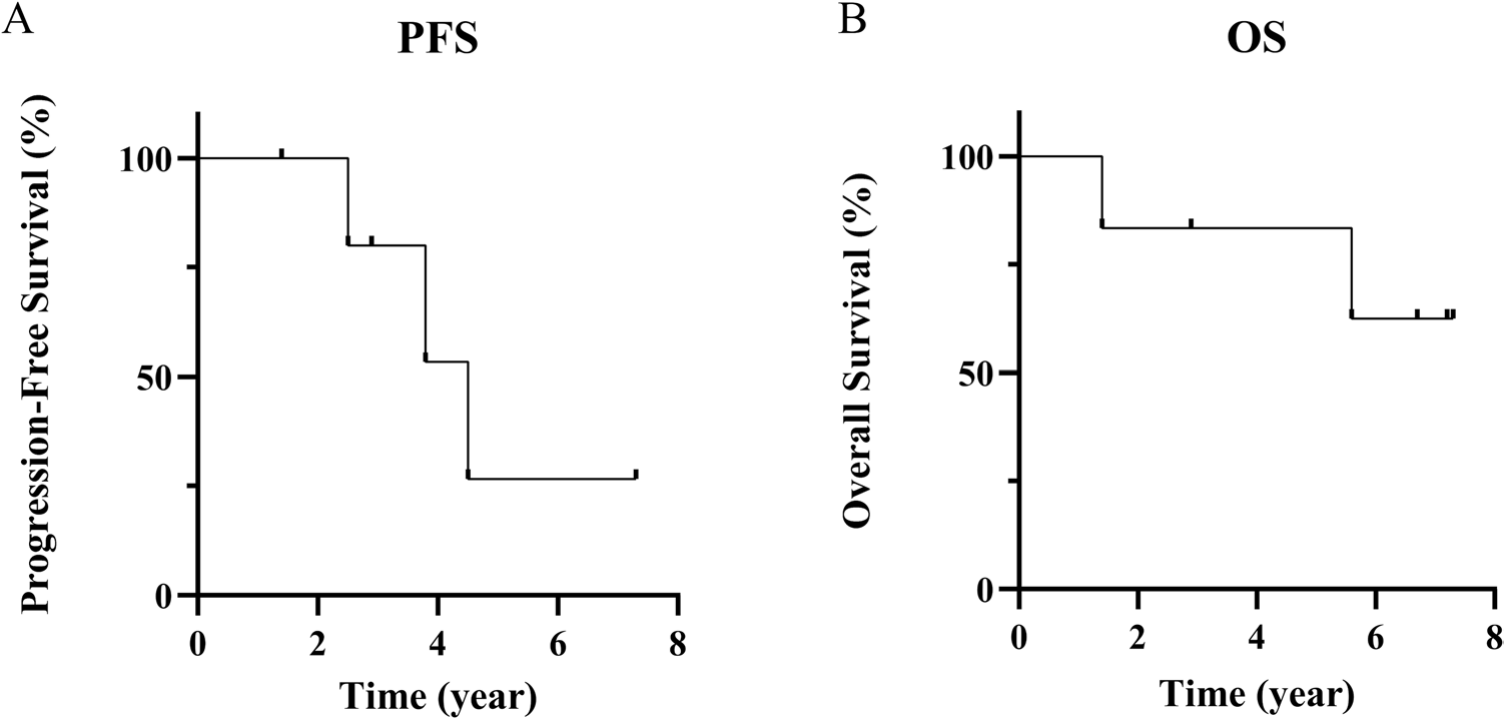
Progression-free survival and overall survival in our cohort. The median progression-free survival (PFS) was 3.4 years (**A**). The median overall survival (OS) was 6.2 years (**B**)

The median overall survival (OS) was 6.2 years (range: 1.4–7.3 years). Three of the six patients had tumor recurrence, and the median progression-free survival (PFS) was 3.4 years (range: 1.4–7.3 years) (Fig. 1A, B). Among the six patients, two died during the observation period: one from acute myeloid leukemia (Case 2) and one from the tumor (Case 4).

### Pathological analysis

Histological diagnosis included three cases of PA, one case of anaplastic astrocytoma, one case of glioblastoma, and one case of astrocytoma according to the fourth edition of the WHO Classification of Tumours of the CNS. Genome-wide DNA methylation profiling was performed for Case 2 and 3, and F1CDx for Case 2 and 6. The integrated diagnosis, according to the fifth edition of the WHO Classification of Tumours of the CNS, included two cases of PA and one case each of diffuse high-grade glioma, not elsewhere classified (NEC); diffuse midline glioma H3 K27-altered; glioblastoma, not otherwise specified (NOS); and circumscribed astrocytic glioma, NOS (Table 1).

### Illustrative cases

#### Case 2

**Fig. 2:**
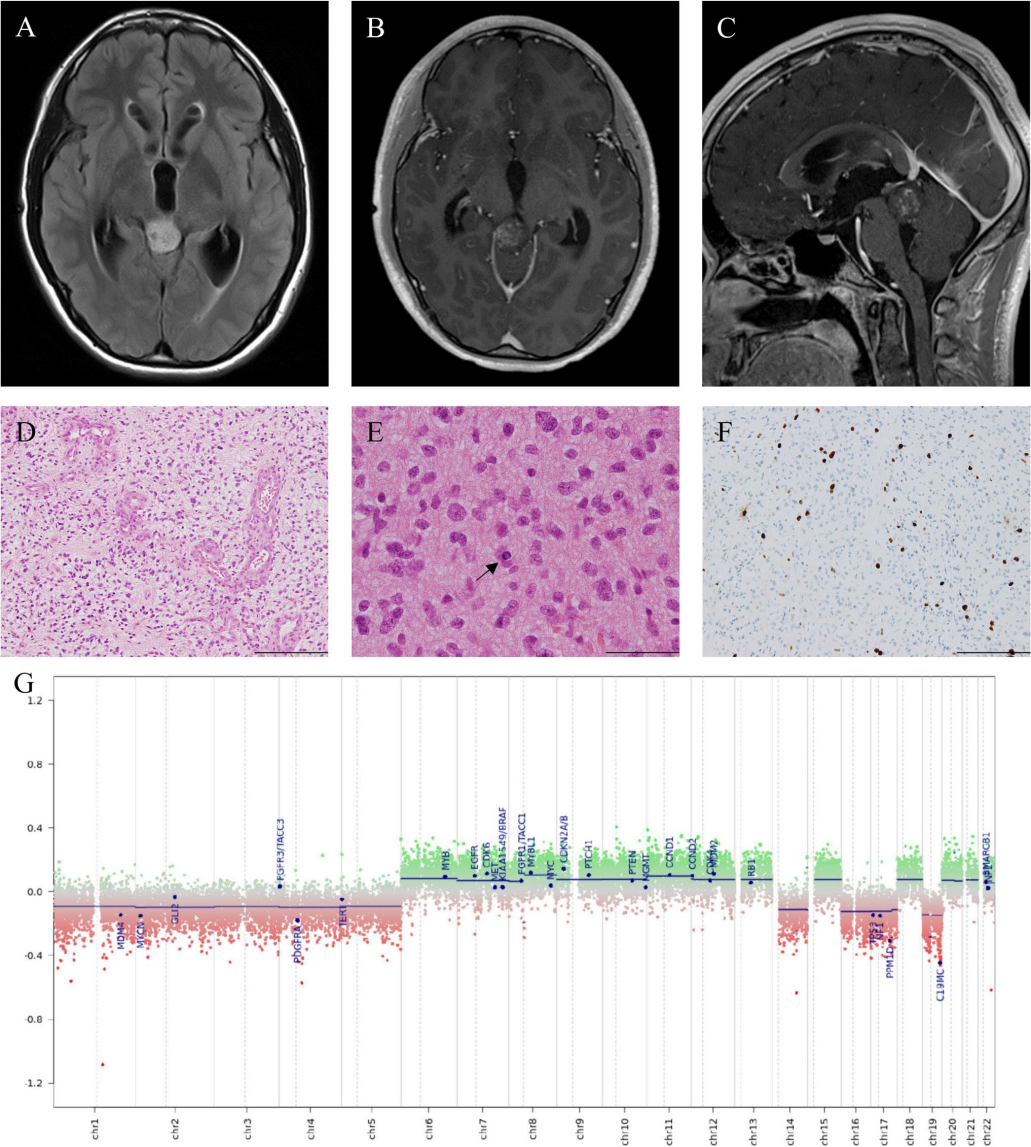
Radiological, histological findings and DNA methylation analysis in Case 2 (**A**–**G**). The tumor was located in the tectum and exhibited high intensity on FLAIR (Fluid Attenuated Inversion Recovery) images (**A**) and partial enhancement after gadolinium administration (**B**, **C**). Hematoxylin and eosin staining revealed microvascular proliferation (**D**), mitosis (with the arrow indicating a mitotic cell), and proliferation of atypical tumor cells with irregular nuclei (**E**). The Ki-67 proliferation index was 5.7% in the most stained areas (**F**). The scale bar indicates 200 μm (**D**, **F**) and 50 μm (**E**). Genome-wide DNA methylation showed no *KIAA1549::BRAF* fusion (**G**)

A 14-year-old Japanese male presented with headaches and vomiting. MRI revealed a 22.7 mm tumor lesion in the tectum with contrast enhancement (Fig. 2A–C). Following emergency ventricular drainage, he was referred to our hospital for treatment. While an endoscopic biopsy, third ventriculostomy, and Ommaya reservoir placement were performed, the surgical specimen was insufficient for a conclusive diagnosis. Given MRI indications of a dual lesion in both the hypothalamus and pineal, coupled with spontaneous regression after X-ray exposure, we clinically diagnosed the case as a germinoma. Subsequently, he underwent radiation therapy (23.4 Gy/13 Fr) and chemotherapy (Carboplatin: 450 mg/m^2^, Etoposide: 150 mg/m^2^). Post-adjuvant therapy MRI showed a residual tumor, leading to tumor resection via an occipital interhemispheric transtentorial approach 4 months after the initial surgery. The histopathological diagnosis was glioblastoma, IDH-wild type, according to the fourth edition of the WHO Classification of Tumours of the CNS. Atypical cells with rounded, enlarged nuclei were densely proliferating. The tumor component exhibited mitotic figures and microvascular proliferation, but no necrosis. Mitosis was observed at approximately one per 10 fields of view at high magnification (Fig. 2D, E). The tumor cells were immunohistochemically positive for GFAP, ATRX, and H3 K27me3, but negative for synaptophysin, IDH1 R132H, and H3 K27M. p53-positive cells made up less than 10%, and the Ki-67 proliferation index was 5.7% in the most stained areas. (Fig. 2F). The genome-wide DNA methylation profile indicated PA by the DKFZ classifier. However, those calibrated scores were 0.71 in v11b4 and 0.60647 in v12.5 (Table 1). Genome-wide copy number plot showed intact copy number of *KIAA1549::BRAF* region (Fig. 2G). Genetic analysis with F1CDx identified *KRAS* G12A and *KRAS* G13R without any known *BRAF* mutations (Table 1). Based on these results, we finally descriptively diagnosed this case as diffuse high-grade glioma, CNS WHO grade 4, not elsewhere classified (NEC) (commonly referred to as TG). The postoperative course was good, leading to the patient’s discharge with no obvious residual neurological symptoms. However, during follow-up, he developed acute myeloid leukemia and alveolar hemorrhage, suspected to be leukemic infiltration. He eventually died 1.4 years after the initial surgery.

#### Case 3

**Fig. 3:**
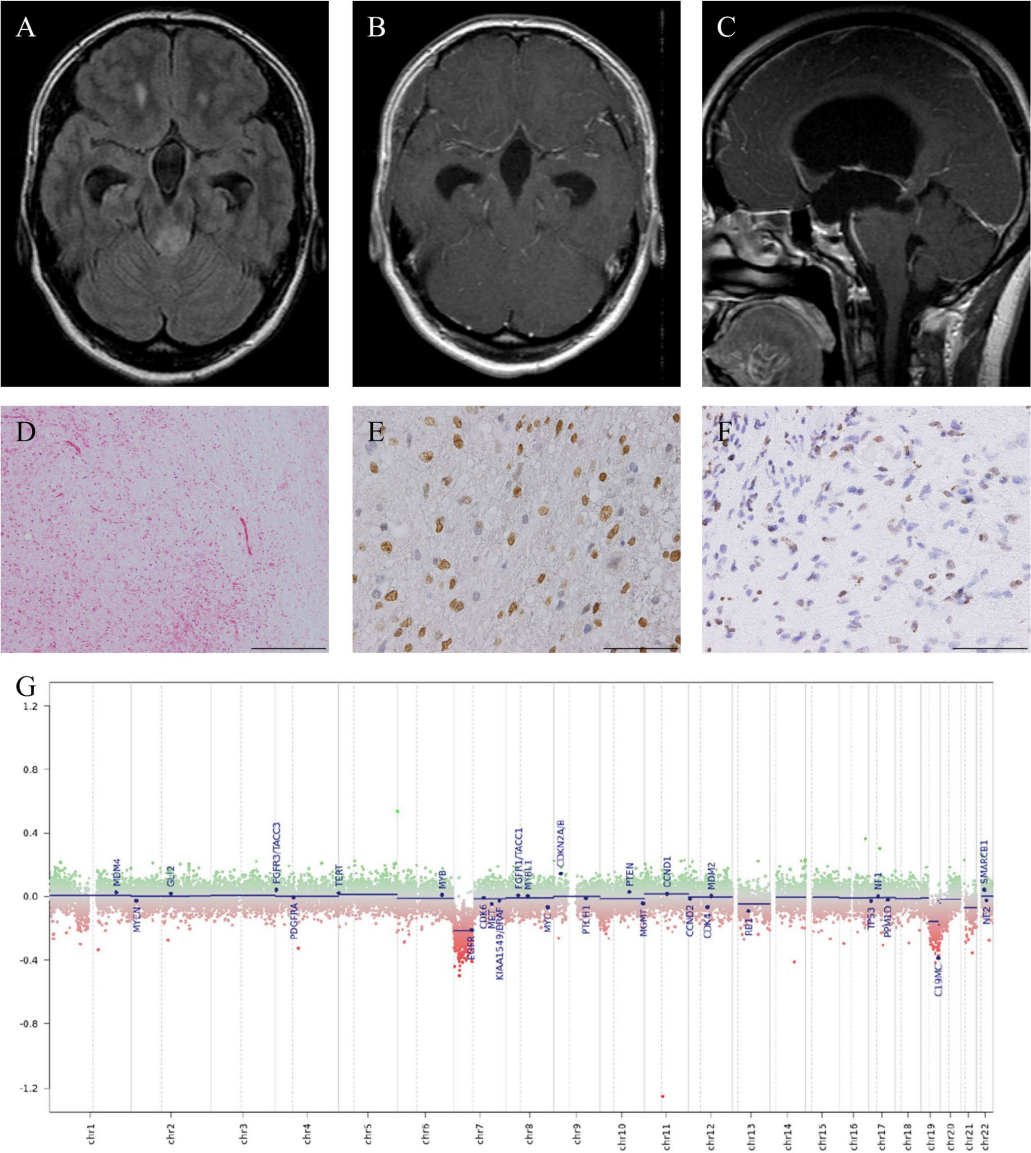
Radiological, histological findings and DNA methylation analysis in Case 3 (**A**–**F**). The ventricles were enlarged, the tumor was located in the tectum and exhibited high intensity on FLAIR images (**A**). Tumor was not enhanced after gadolinium administration (**B**, **C**). Hematoxylin and eosin staining revealed a biphasic pattern with alternating dense and loose components (**D**). Glial cells with long, hair-like (piloid) processes were observed, and Rosenthal fibers appeared as brightly eosinophilic structures (**D**). Tumor cells were positive for the H3K27M antibody (**E**) and negative for the H3K27me3 antibody (**F**) in immunohistochemistry. The scale bar indicates 200 μm (**D**) and 50 μm (**E**, **F**). The copy-number profile of the genome-wide DNA methylation is shown (**G**)

A 40-year-old Japanese female was incidentally diagnosed with hydrocephalus through MRI. Four years later, she was referred to our department due to headaches. MRI revealed a 16.4 mm tumor lesion in the tectum without contrast enhancement (Fig. 3A–C). Subsequently, she underwent endoscopic third ventriculostomy and biopsy, with the pathological diagnosis indicating PA. She received no further treatment, but experienced memory impairment and gait disturbance 45 months after the initial surgery, and MRI revealed tumor enlargement. A second endoscopic third ventriculostomy and biopsy were performed, confirming the persistence of PA. Postoperatively, her neurological symptoms improved. However, 60 months after the initial surgery, she presented with gait disturbance and ataxia, and an MRI revealed tumor recurrence. A stereotactic biopsy was performed, yielding the pathological diagnosis of PA. The tumors exhibited a biphasic pattern with alternating dense and loose components. Glial cells with long, hair-like (piloid) processes were observed, and Rosenthal fibers appeared as brightly eosinophilic structures (Fig. 3D). Despite undergoing postoperative radiation therapy (50 Gy/25 Fr), her gait disturbance and urinary incontinence continued to worsen. Eventually, 83 months after her initial surgery, she was transferred to palliative care. We retrospectively performed DNA methylation profiling using DNA extracted from frozen tissue from the third surgery. Despite the histological diagnosis of PA, the DNA methylation profile in DKFZ classifier v11b4 suggested a diffuse midline glioma (DMG) with H3 K27M mutation, calibrated with a score of 0.93008 and DMG with H3 K27M mutation in v12.5 with calibrated score of 0.67318. (Table 1). Immunohistochemistry also confirmed the presence of *H3-3A* K27M mutation and loss of H3 K27me3 (Fig. 3E, F). We finally diagnosed this case as DMG H3 K27-altered, CNS WHO grade 4.

## Discussion

In this study, we conducted a retrospective review about the clinical, radiological, pathological, and molecular characteristics of TG cases in our institute. Although the clinical course of TG which is mostly diagnosed as PA or LrGG is typically indolent, the median PFS and OS were 3.4 years and 6.2 years, respectively, in our series. Previous reports showed that the clinical course of typical PA after a gross total resection is usually favorable, with a 10-year overall survival (OS) rate of approximately 95% [2, 15, 26, 28]. However, Liu et al. [12] reported that 121 of 453 (26.7%) TG patients experienced clinical and/or radiological progression, with the mean time from diagnosis to progression ranging from 3 months to 7.8 years. Thus, these findings suggested that TG may exhibit relatively poorer PFS and OS compared to typical PA or LrGGs in other locations and TG is not always an indolent tumor.

Regarding the pathological analysis, our study identified that TG included diverse diagnosis such as pilocytic astrocytoma, glioblastoma, diffuse high-grade glioma, and DMGs H3 K27-altered. Previously the histopathological findings of TG are typically associated with LrGG such as PA or diffuse astrocytoma. In a systematic review of 355 pediatric TG patients, Bauman et al. [1] reported that, among 117 pathologically classifiable cases, 37.6% had PA, 25.6% had diffuse astrocytoma, and 12.8% had low-grade (non-pilocytic) glioma. In contrast, Mohme et al. [19] reported that in 12 of 23 pathologically classifiable cases, 58.3% were LrGG (5 PA, 1 ganglioglioma, and 1 diffuse astrocytoma), while 42.7% were classified as HGGs (3 glioblastomas, and 2 anaplastic astrocytomas). Other reported pathological findings of TG include morphologically PA with genetic H3 K27M mutation, pleomorphic xanthoastrocytoma, and atypical teratoid/rhabdoid tumors [20, 21, 25, 27, 30]. These findings suggest that TG has heterogeneous pathology, despite being predominantly an LrGG.

Regarding the molecular analysis, the *KRAS* mutations and *BRAF* alterations have recently been observed in TG. In addition, the genome-wide DNA methylation profile indicates that TG is a distinct tumor in comparison to other LrGGs [6, 8, 11, 12]. Chiang et al. [6] reported a high frequency of *KRAS* G12R mutations (82.6%) and *BRAF* mutations (60.9%) in their analysis of 23 TG cases. In addition, other reported mutations in TG include *KRAS* p.E63K missense mutation and *KRAS* c181C>A mutation [5, 31, 32]. Liu et al. [12] conducted genome-wide DNA methylation analysis in 45 cases of TG, revealing a distinct methylation pattern compared to PAs at other sites. In our study, two patients (Case 2 and 6) underwent F1CDx, and both harbored *KRAS* alterations (*KRAS* G12A and *KRAS* G13R in Case 2, and *KRAS* Q61R in Case 3), which suggested that there may be a class of TGs with *KRAS* mutation. The molecularly evaluated TG reports from the last decade are summarized in Table 2. *KRAS* mutations and *BRAF* alterations have been reported relatively frequently in TG with LrGG such as PA and DA, while a few mutations like H3 K27M and *SMARCB1* have been observed less frequently. This study also identified *KRAS* and H3 K27M mutations in two cases histologically confirmed with LrGG (Table 2).

**Table 2:** Summary of literature on tectal glioma with molecular analysis

Authors	Year	N	Age at diagnosis (range)	Male	Pathology	Molecular analysis	Biopsy/resection	Radiotherapy	Chemotherapy	Progressive disease	Follow-up (range)
Wu et al. [30]	2016	1	51 years	1	AT/RT = 1	SMARCB1/INI1 RHPN2L385I, MDM4D396G, FLT3 V194M, NPRL3D53N	Bx then 2nd resection	54 Gy	Temozolomide	no	12 months
Liu et al. [12]	2018	45	Median: 9.9 years (0.01–20.5)	26	Similar to PA = 25 Similar to DA = 5 30 Available to pathology	KIAA1549-BRAF fus (6/24) BRAF V600E mut (2/26)	7 (32%) Bx = 3 GTR = 3 Partial resection (spinal metastasis) = 1	5 (54–55.8 Gy)	4	7 (32%) Median time to progression: 0.68 year (0.28–8.98)	Median: 7.64 years (0.51–16.98)
Mohme et al. [19]	2018	23	Mean: 29.2 years (1–69)	12	PA = 5 DA = 1 GG = 1 GBM = 3 AA = 2 12 available to pathology	H3K27M = 1 (1/12)	12 (52.2%) Resection = 6 Bx = 3 Bx then 2nd resection = 3	3	3	Mean: 51.5 months	Mean: 73 months (7–429)
Morita et al. [20]	2018	1	53 years	1	PA	H3K27M	Resection	no	no	no	8 months
Chau et al. [5]	2019	1	24 years	0	PA	KRAS c187G>A (KRAS pE63K)	Bx	no	no	7 years	16 years
Nagaishi et al. [21]	2020	1	47 years	0	PXA	BRAF alternation (KIAA1549-BRAF/V600E)	Resection	54 Gy	Temozolomide	5 months	5 months
Chiang et al. [6]	2020	23	Median: 11 years (2–21)	10	N/A	KRASG12R (19/23) BRAF alternation (KIAA1549-BRAF/V600E/T599dup) (14/23)	N/A	N/A	N/A	N/A	N/A
Sumi et al. [25]	2020	1	13 years	0	PA	H3K27M	Bx then 2nd resection	50 Gy	Temozolomide	no	28 months
Wulfovich et al. [31]	2021	1	8 years	1	PA	KRAS c181C>A (KRAS pQ61K)	Bx	no	no	no	36 months
Yakir et al. [32]	2023	4	Median: 5.5 years (1–8)	1	PA = 3 PMA = 1	KRAS c181C>A = 1 KRAS c187G>A = 1 EGFR R222C = 1 SRGAP3-RAF-1 fusion = 1	Bx = 4	N/A	2	N/A	Median: 6 years (4–20)

*AA* anaplastic astrocytoma, *AT/RT* atypical teratoid/rhabdoid tumor, *Bx* biopsy, *DA* diffuse astrocytoma, *fus* fusion, *GBM* glioblastoma, *GG* ganglioglioma, *GTR* gross total resection, *N* number, *N/A* not applicable or data not available, *mut* mutation, *PA* pilocytic astrocytoma, *PMA* pilomyxoid astrocytoma, *PXA* pleomorphic xanthoastrocytoma

Although RAS mutations are generally rare in gliomas and not associated with a specific tissue phenotype [17], a relatively large proportion of TGs have been reported *KRAS* mutations and *BRAF* alterations. *KRAS* is involved in several signaling cascades, including MAPK, PI3K/ACT, RAL-GDS, JAK/STAT3, and NORE1/RASSF1 pathways, influencing various cellular processes such as growth, apoptosis, growth arrest, differentiation, transcription, and translation [5, 17, 22, 31]. For example, *KRAS* signaling is essential for the maintenance of glioblastoma growth maintenance in mice, suggesting that inhibition of *KRAS* leads to tumor apoptosis [3, 7]. In addition to hotspot mutations in *KRAS*, Yakir et al. [32] reported *SRGAP3::RAF1* fusion in pediatric TG. *SRGAP3::RAF1* fusion has also been reported in pediatric PA cases and abnormally activates both MAPK and PI3K/mTOR signaling pathways [9, 23]. These findings support that there is a group of TGs with mutations in *KRAS* or its downstream pathway, exhibiting a distinct methylation pattern and differing entity from other LrGGs. However, the detailed molecular landscape of *KRAS* alterations in TG remains unclear, and further analysis is required.

In our study, Case 3 was pathologically diagnosed as PA, and H3 K27M mutation was inferred by genome-wide DNA methylation profiling and immunohistochemistry (Fig. 3D–F). We have identified three reports of TG with histologic findings of PA and H3 K27M mutation, including our case [20, 25]. In the 2021 WHO Classification of Tumors of the Central Nervous System, DMGs were renamed DMGs H3 K27-altered, considering the various mechanisms affecting the epigenetic pathway [13, 14, 24]. Conversely, several non-DMG tumors, such as ependymoma, PA, pediatric diffuse astrocytoma, and ganglioglioma, have been reported to harbor the same H3 K27M mutation, displaying a different clinical presentation and a favorable prognosis compared to typical DMGs H3 K27-altered [13, 20, 25]. Sumi et al. described these as nontypical DMGs. Two reported cases of morphologic PA with H3 K27M mutation showed no recurrence during observation periods of 8 and 28 months, respectively (Table 2). Case 3 had a PFS of 4.5 years and an OS of 7.2 years (Table 1). These results suggest that PAs with H3 K27M mutations tend to have a relatively better prognosis compared to typical DMG H3 K27-altered patients. We consider the PA with H3 K27M mutation in our study to be a part of this group, often referred to as nontypical DMGs.

We summarized previous reports of TGs that have been evaluated molecularly in Table 2. Given the diverse histological and molecular features of TG, there is a potential discrepancy between histological and molecular evaluations. Thus, TG requires detailed histological and molecular analysis including genome-wide DNA methylation, and further accumulation of knowledge is needed to establish this pathogenesis as a clear entity.

Limitations of this study include its retrospective design, small number of cases, and the lack of genetic information in some cases. In addition, although we included only symptomatic cases in this study, evaluating asymptomatic cases with an untreated imaging course is considered necessary.

## Conclusion

Our analysis revealed that TG exhibits diverse histological and molecular characteristics, distinguishing it from other LrGGs. Although TG is generally considered to be an indolent tumor, there exists a potential risk of disease progression throughout the clinical course. Given the heterogeneous pathological background of TG and the associated risk of pathological progression, we emphasize the importance of a comprehensive diagnosis, including molecular evaluation.

## Data Availability

The datasets generated and/or analyzed during the current study are available from the corresponding author on reasonable request.

## Abbreviations

DMGs: Diffuse midline gliomas
F1CDx: FoundationOne^®^ CDx
HGG: High-grade glioma
LrGG: Lower grade glioma
MRI: Magnetic resonance imaging
OS: Overall survival
PA: Pilocytic astrocytoma
PFS: Progression-free survival
RANO: Response Assessment in Neuro-Oncology
TG: Tectal glioma
